# Long-term thyroid outcomes and survival in hyperthyroid cats treated with radioiodine: association with overt and subclinical hypothyroidism and levothyroxine treatment

**DOI:** 10.1093/jvimsj/aalag233

**Published:** 2026-09-26

**Authors:** Mark E Peterson, Stephanie Carmody, Mark Rishniw

**Affiliations:** Animal Endocrine Clinic, 21 West 100th Street, New York, NY, United States; College of Veterinary Medicine, Cornell University, Ithaca, NY, United States; Animal Endocrine Clinic, 21 West 100th Street, New York, NY, United States; College of Veterinary Medicine, Cornell University, Ithaca, NY, United States; Veterinary Information Network, Davis, CA, United States

**Keywords:** CKD, creatinine, feline, hypothyroidism, radioactive iodine, survival, thyroid gland

## Abstract

**Background:**

Long-term thyroid status and survival after radioiodine treatment of hyperthyroid cats are incompletely characterized, particularly for cats developing iatrogenic hypothyroidism.

**Hypothesis/Objectives:**

To determine long-term thyroid outcomes and survival after radioiodine treatment, and to assess associations among thyroid status, hypothyroid remission, levothyroxine treatment, and survival.

**Animals:**

One thousand three hundred forty-four hyperthyroid cats treated with radioiodine.

**Methods:**

Prospective longitudinal cohort study. Thyroid and renal status were classified 6-12 months after radioiodine treatment and reassessed at 6- to 12-month intervals until death, euthanasia, relapse, loss to follow-up, or study completion. Kaplan–Meier analysis and Cox proportional hazards regression evaluated survival.

**Results:**

At 6-12 months, 1047/1344 cats (77.9%) were euthyroid, 240/1344 (17.9%) were subclinically hypothyroid, and 57/1344 (4.2%) were overtly hypothyroid. Among initially euthyroid cats, 934/1047 (89.2%) remained euthyroid, 43 (4.1%) developed late-onset hypothyroidism, and 70 (6.7%) developed recurrent hyperthyroidism. Among initially subclinically hypothyroid cats, 102/240 (42.5%) underwent remission to become euthyroid. Median survival was 49.7 months in euthyroid cats, 44.6 months in subclinically hypothyroid cats, and 36.1 months in overtly hypothyroid cats. In multivariable analysis, initial posttreatment thyroid status was not independently associated with survival, whereas older age, male sex, and higher creatinine concentration were. In final-outcome models, untreated subclinical and overt hypothyroidism were independently associated with an increased hazard of death, whereas hypothyroidism in cats treated with levothyroxine was not.

**Conclusions and clinical importance:**

Thyroid status after radioiodine treatment is dynamic. Subclinical hypothyroidism frequently resolves, whereas untreated persistent hypothyroidism is associated with shorter survival. Long-term monitoring of thyroid and renal function is critical in managing radioiodine-treated cats.

## Introduction

Hyperthyroidism is the most common endocrine disorder in cats. Most clinicians consider radioactive iodine (radioiodine; ^131^I) treatment to be the gold standard, with an efficacy of over 95% in hyperthyroid cats.^1^^,^^2^ However, despite its widespread use for over 4 decades, limited information exists on the long-term outcomes or survival rates of ^131^I-treated cats, with most studies focusing on cure rates within the first 6-12 months of treatment.^3–19^ Furthermore, important aspects of iatrogenic hypothyroidism after ^131^I treatment remain poorly characterized, including whether ^131^I-induced hypothyroidism is invariably permanent or if it can resolve as residual normal (but atrophied) thyrocytes recover and resume functioning.^10^^,^^16^ Similarly, investigators have given little attention to differentiating between mild (subclinical) and severe (overt) forms of hypothyroidism in cats or to determining whether either of these hypothyroid subgroups would benefit from thyroid hormone supplementation. Understanding these variables is crucial for tailoring treatment plans or predicting long-term outcomes or survival.

In this study, we sought to explore several factors influencing the long-term thyroid status and survival in a large cohort of hyperthyroid cats treated with ^131^I. Our first objective was to evaluate the long-term thyroid status of cats treated with ^131^I, focusing on whether cats classified as euthyroid at 6-12 months remain euthyroid for the rest of their lives or if some develop late-onset hypothyroidism or relapse of hyperthyroidism, and the frequency of these outcomes. The second aim was to determine whether cats classified as hypothyroid (overt or subclinical) at 6- to 12-month intervals remain hypothyroid or if they can transition to become euthyroid or experience relapse, and the frequency of such remission and relapse. Our third objective was to assess whether survival times vary based on short-term outcomes by comparing the survival rates of cats categorized as euthyroid, subclinically hypothyroid, or overtly hypothyroid at 6-12 months after ^131^I treatment. The fourth objective was to determine the association of age, sex, and development of azotemic chronic kidney disease (CKD) with survival. Finally, our study sought to determine whether levothyroxine supplementation improves survival in hypothyroid cats, particularly those with severe (overt) hypothyroidism.

## Materials and methods

This study included 1344 hyperthyroid cats successfully treated with ^131^I. Clinical details, including the ^131^I dosing algorithm and 6- to 12-month follow-up data, were previously reported as part of a 1400-cat cohort.^20^ Cats were enrolled from January 2013 through June 2020, and follow-up continued through January 2025.

After thyroid and renal status were initially classified at 6-12 months (Table 1),^20^ cats were reevaluated at 6- to 12-month intervals until death, euthanasia, loss to follow-up, or study completion. At each recheck, a physical examination was performed and serum thyroxine (T4), thyroid-stimulating hormone (TSH), and creatinine concentrations were measured. For cats not reexamined at our clinic, follow-up laboratory and survival data were obtained from the referring veterinarian and owner. Thyroid status was reclassified as needed at each reevaluation based on serum T4 and TSH concentrations. For cats that developed overt or subclinical hypothyroidism, levothyroxine supplementation was offered at the discretion of the owner and referring veterinarian, with the goal of normalizing serum T4 and TSH concentrations.^21^^,^^22^ Untreated hypothyroid cats were monitored for spontaneous return to euthyroidism.^22^ Final thyroid status was recorded at death or last follow-up.

**Table 1:** Classification of thyroid outcome status into euthyroid, overt hypothyroid, and subclinical hypothyroid groups at 6-12 months in 1344 ^131^I-treated cats.

**Variable**	**All cats (1344)**	**Euthyroid (1047)**	**Overt hypothyroid (57)**	**Subclinical hypothyroid (240)**	***P* value**
**Age (years)**	12 (10-14)	12^a,b^ (10-14)	13^a^ (12-15)	13^b^ (11-14)	<.0001^*^
**Breed (mixed: purebred ratio)**	1204:140 (8.6)	930:117 (8.0)	52:5 (10.4)	222:18 (12.3)	.22^†^
**Sex ratio (female:male)**	719:625 (1.15)	521:526 (0.99)^a,b^	41:16 (2.56)^a^	157:83 (1.90)^b^	<.0001^†^
**Serum T4 (μg/dL)**	1.7 (1.4-2.1)	1.9^a,b,c^ (1.5-2.3)	0.7^a,c^ (0.5-0.7)	1.4^a,b^ (1.2-1.7)	<.0001^*^
**Serum TSH (ng/mL)**	0.11 (0.03-0.29)	0.07^a,b,c^ (0.03-0.14)	4.65^a,c^ (2.44-9.8)	1.08^a,b^ (0.56-2.7)	<.0001^*^
**Serum creatinine (mg/dL)**	1.6 (1.3-2.0)	1.6^a,b,c^ (1.3-1.9)	2.3^a,c^ (2.0-2.7)	1.9^a,b^ (1.5-2.4)	<.0001^*^
**Azotemic (no. of cats)**	262 (19.5%)	128 (12%)	40 (70%)	94 (39%)	<.0001^†^

Each cat’s thyroid status was classified into 1 of 3 categories based on the serum T_4_ and TSH concentrations measured at 6-12 months after ^131^I treatment: euthyroid (T_4_ = 1.0-3.8 μg/dL; TSH ≤ 0.30 ng/mL); overtly hypothyroid (T_4_ < 1.0 μg/dL; TSH > 0.30 ng/mL); or subclinically hypothyroid (T_4_ = 1.0-3.8 μg/dL; TSH > 0.30 ng/mL). Cats were also classified as being azotemic or nonazotemic, with renal azotemia defined as a serum creatinine concentration > 2.0 mg/dL.

Continuous data are expressed as median (25th-75th percentile), whereas qualitative data are expressed as ratio (breed, sex).

a, b, c: Values within a row with the same superscript letters are significantly different from one another.

^*^Kruskal–Wallis test, followed by Dunn multiple comparisons test.

^†^χ^2^ test, followed by the Holm–Bonferroni correction procedure for within group comparison.

Abbreviations: T_4_ = thyroxine; TSH; thyroid-stimulating hormone.

Survival time was defined as the interval from ^131^I treatment to death or euthanasia. Cats alive at study completion and cats lost to follow-up were right-censored at their last examination.^23^^,^^24^ Cats were considered lost to follow-up if they had not been evaluated for > 6 months and the owner could not be contacted.

Statistical analyses were performed with GraphPad Prism version 11.0 (GraphPad Software, La Jolla, CA). Continuous variables were reported as median, IQR, and CI. Statistical significance was set at *P* ≤ .05. Multivariable logistic regression was used to identify factors associated with remission of hypothyroidism and relapse of hyperthyroidism.

For survival analysis, cats were grouped according to thyroid status at 6-12 months and at study end. Hypothyroid cats were further grouped by levothyroxine treatment. An additional analysis included an expanded cohort of all cats that developed overt hypothyroidism at any time during follow-up; for this cohort, survival time was calculated from diagnosis of overt hypothyroidism to death or censoring. Kaplan–Meier curves were generated and compared by log-rank test.^23^^,^^25^ Multivariable Cox proportional hazards regression was used to determine whether thyroid status or levothyroxine treatment was independently associated with survival after adjustment for age, sex, and serum creatinine concentrations.^26^^,^^27^ Additional statistical details are provided in the Appendix.

## Results

### Characteristics of the ^131^I-treated cat cohort

**Figure 1:**
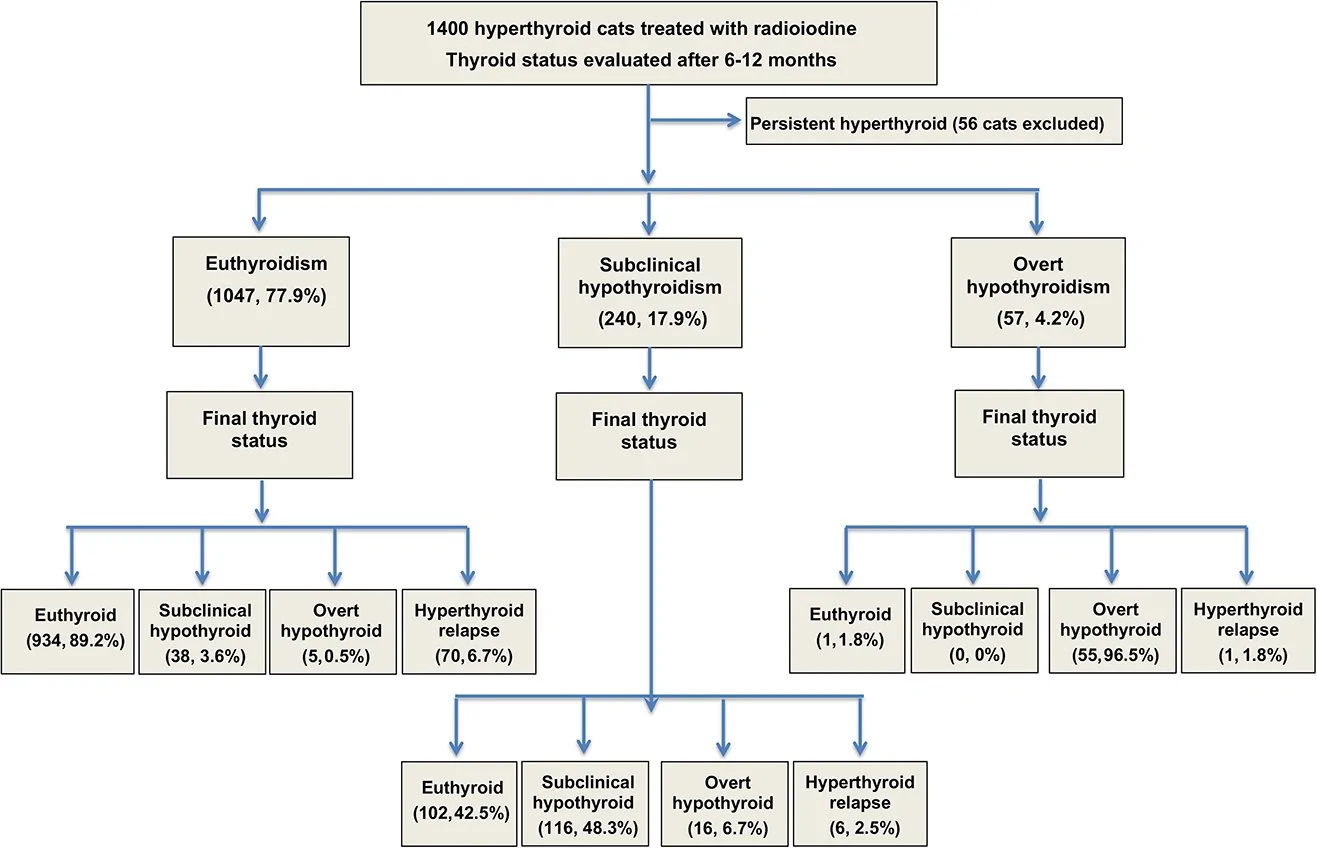
Flowchart indicating the long-term thyroid status and outcome of 1344 cats treated with radioiodine. At 6-12 months after treatment, cats were classified as euthyroid, subclinically hypothyroid, or overtly hypothyroid.^20^ Thereafter, thyroid status was reclassified as needed on the basis of follow-up serum T4 and TSH concentrations measured at 6- to 12-month intervals until death or last follow-up. Abbreviations: T4, thyroxine; TSH, thyroid-stimulating hormone.

Of the 1344 hyperthyroid cats treated with radioiodine, 1047 (77.9%) became euthyroid, 57 (4.2%) developed overt hypothyroidism, and 240 (17.9%) developed subclinical hypothyroidism at 6-12 months (Table 1; Figure 1). Cats that developed overt or subclinical hypothyroidism were older than the euthyroid cats, and a greater proportion were female (Table 1).

Hypothyroid cats had lower posttreatment serum T4 concentrations and higher serum TSH and creatinine concentrations than euthyroid cats. Cats with overt hypothyroidism also had lower serum T4 concentrations and higher serum TSH and creatinine concentrations than cats with subclinical hypothyroidism (Table 1).

### Long-term outcome of thyroid status in 1344 cats treated with radioiodine

#### Cats classified as euthyroid at 6-12 months

Of the 1047 cats classified as euthyroid at 6-12 months, 934 (89.2%) remained euthyroid at the end of the study (Figure 1). Of the remaining 113 cats, 43 subsequently developed late-onset subclinical (*n* = 38) or, less commonly, overt (*n* = 5) hypothyroidism, whereas 70 (6.7%) developed relapse of hyperthyroidism (Figure 1).

The time to development of late-onset overt/subclinical hypothyroidism in the 43 cats ranged from 13 to 48 months (median, 15 months; IQR, 14-22 months). Risk factors for late-onset hypothyroidism included older age (*P* = .007; odds ratio, 1.21; 95% CI, 1.05-1.40), lower posttreatment serum T4 concentration (*P* = .041; odds ratio, 2.0; 95% CI, 1.03-3.94), and higher posttreatment serum TSH concentration at 6-12 months (*P* < .001; odds ratio, 1.95; 95% CI, 1.38-2.77). Twenty-six (60%) of these cats were azotemic at the time of diagnosis of late-onset hypothyroidism.

The time from ^131^I treatment to relapse of hyperthyroidism in the 70 cats that were initially euthyroid ranged from 23 to 84.2 months (median, 47.1 months; IQR, 36-57 months). Risk factors for relapse in ^131^I-treated euthyroid cats included younger age at diagnosis (*P* = .0058; odds ratio, 0.86; 95% CI, 0.77-0.96), higher posttreatment serum T4 concentration at 6-12 months (*P* = .0011; odds ratio, 1.90; 95% CI, 1.29-2.78), and a longer interval since ^131^I treatment (>3 years; *P* = .016; odds ratio, 2.80; 95% CI, 1.21-6.26).

#### Cats classified as overtly hypothyroid at 6-12 months

Almost all cats (55/57; 97%) classified as having overt hypothyroidism (low serum T4 with high TSH concentrations^28^) at 6-12 months remained overtly hypothyroid during follow-up (Figure 1). One cat became euthyroid and 1 developed recurrent hyperthyroidism at 60.1 and 50.3 months, respectively, after ^131^I treatment (Figure 1).

Of the 57 cats with overt hypothyroidism, 46 received levothyroxine treatment and 11 did not. Levothyroxine treatment was generally instituted 1-2 months after diagnosis, once repeat testing confirmed persistently low serum T4 concentrations and markedly increased serum TSH concentrations (Table 1). All overtly hypothyroid cats had serum TSH concentrations > 1.5 ng/mL at diagnosis, and 46 (81%) had TSH concentrations > 2.5 ng/mL. Levothyroxine treatment normalized serum T4 concentrations (median, 2.4 μg/dL; IQR, 2.2-2.9 μg/dL) and markedly decreased serum TSH concentrations in all treated cats (median, 0.19 ng/mL; IQR, 0.04-0.51 ng/mL).

#### Cats classified as subclinically hypothyroid at 6-12 months

Of the 240 cats classified as having subclinical hypothyroidism (low-normal serum T4 with high TSH concentrations^28^) at 6-12 months, 116 (48%) remained subclinically hypothyroid, 16 (7%) progressed to overt hypothyroidism, and 108 (45%) had remission of hypothyroidism (Figure 1). Of the 132 cats that either remained subclinically hypothyroid or progressed to overt hypothyroidism, 43 (32.6%) received levothyroxine treatment and 89 did not. Of the 108 cats in which hypothyroidism resolved, 102 (94.4%) reverted to euthyroidism and 6 (5.6%) developed recurrent hyperthyroidism (Figure 1).

In the 108 cats with remission of subclinical hypothyroidism, the time to return to euthyroidism ranged from 10 to 74 months (median, 24 months; IQR, 20-31 months). Cats with remission of subclinical hypothyroidism had lower serum TSH concentrations at 6-12 months than did cats that remained hypothyroid (median, 0.65 ng/mL; IQR, 0.47-1.31 ng/mL vs median, 2.23 ng/mL; IQR, 1.15-4.91 ng/mL; *P* < .001). Predictors of remission of subclinical hypothyroidism included longer survival time per 12 months (odds ratio, 1.54; 95% CI, 1.26-1.87, *P* < .0001), and serum TSH concentration ≤ 1.5 ng/mL (odds ratio, 4.34; 95% CI, 2.35-8.01; *P* < .001).

Among the 7 hypothyroid cats that developed recurrent hyperthyroidism (6 subclinical, 1 overt), the time to relapse ranged from 50.3 to 106 months (median, 58.3 months; IQR, 55.8-66.1 months). This interval was longer than the time to relapse in the euthyroid cats (median, 47.1 months; IQR, 36.2-56.9 months; *P* = .006). In addition, relapse was less common in hypothyroid cats than in euthyroid cats (7/297 [2.4%] vs 70/1047 [6.7%]; *P* = .003).

### Survival of 1344 cats treated with radioiodine

#### Comparison of survival in cats based on thyroid status at 6-12 months after ^131^I treatment

**Figure 2:**
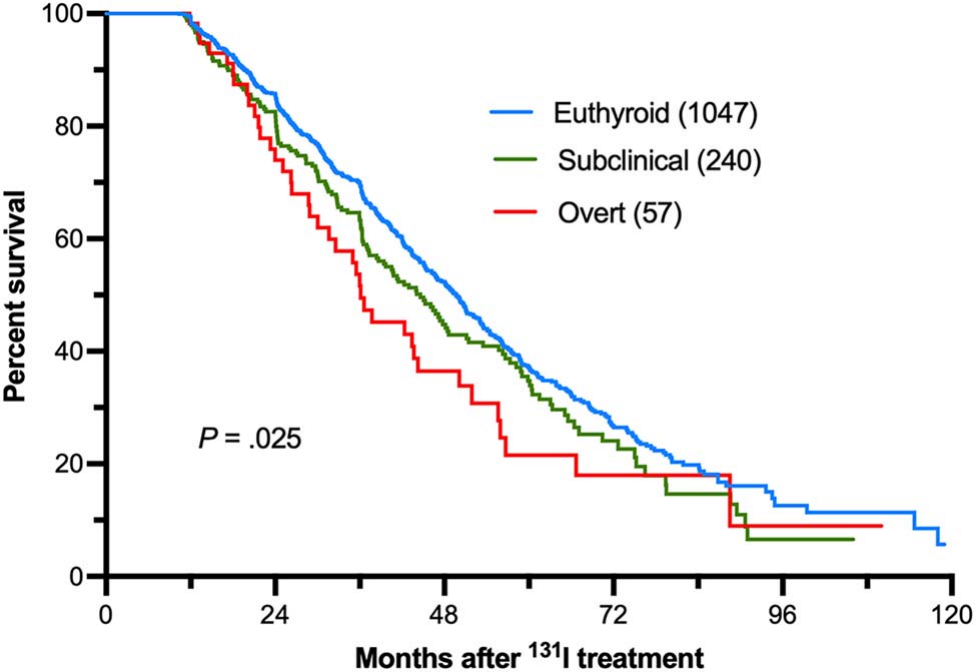
Kaplan–Meier survival curves of 1344 cats treated for hyperthyroidism with radioiodine. Cats were grouped according to thyroid status at the 6-month follow-up period, with survival curves plotted for each group (euthyroid = 1047; subclinical hypothyroid = 240; and overt hypothyroid = 57).

Euthyroid cats had a survival time (median, 49.7 months; 95% CI, 46.7-51.7 months) similar to subclinically hypothyroid cats (44.6 months; 95% CI, 38.4-48.6 months; *P* = .060) but longer than overtly hypothyroid cats (36.1 months; 95% CI, 28.9-44.2 months; *P* = .027; Figure 2). Survival also did not differ between cats with subclinical or overt hypothyroidism (*P* = .262; Figure 2).

In univariable Cox proportional hazards analysis, overt hypothyroidism was associated with an increased hazard of death compared with euthyroidism, whereas subclinical hypothyroidism was not (Table 2). In multivariable Cox proportional hazards analysis adjusting for thyroid group, age, sex, and posttreatment creatinine concentration, thyroid group was no longer independently associated with survival. However, older age, male sex, and higher serum creatinine concentration remained independent predictors of death (Table 2).

**Table 2:** Cox proportional hazards models for survival in 1344 cats classified as euthyroid, subclinically hypothyroid, or overtly hypothyroid at 6-12 months after ^131^I treatment.

**Variable**	**Univariable HR (95% CI)**	***P* value**	**Multivariable HR (95% CI)**	***P* value**
**Subclinical hypothyroid vs euthyroid**	1.18 (0.99-1.41)	.063	1.09 (0.90-1.31)	.383
**Overt hypothyroid vs euthyroid**	1.44 (1.04-1.99)	.028	1.30 (0.93-1.82)	.120
**Age, per 1-year increase**	1.24 (1.20-1.28)	<.0001	1.22 (1.18-1.26)	<.0001
**Male vs female**	1.30 (1.14-1.49)	.0002	1.36 (1.18-1.57)	<.0001
**Creatinine, per 1-mg/dL increase**	1.61 (1.41-1.84)	<.0001	1.29 (1.11-1.49)	.0007

HRs > 1.0 indicate increased hazard of death. Reference groups: thyroid group = euthyroid; sex = female.

The multivariable model included thyroid group, age, sex, and creatinine concentration.

Abbreviation: HR = hazard ratio.

#### Comparison of survival of cats with overt hypothyroidism based on treatment with levothyroxine or not

**Figure 3:**
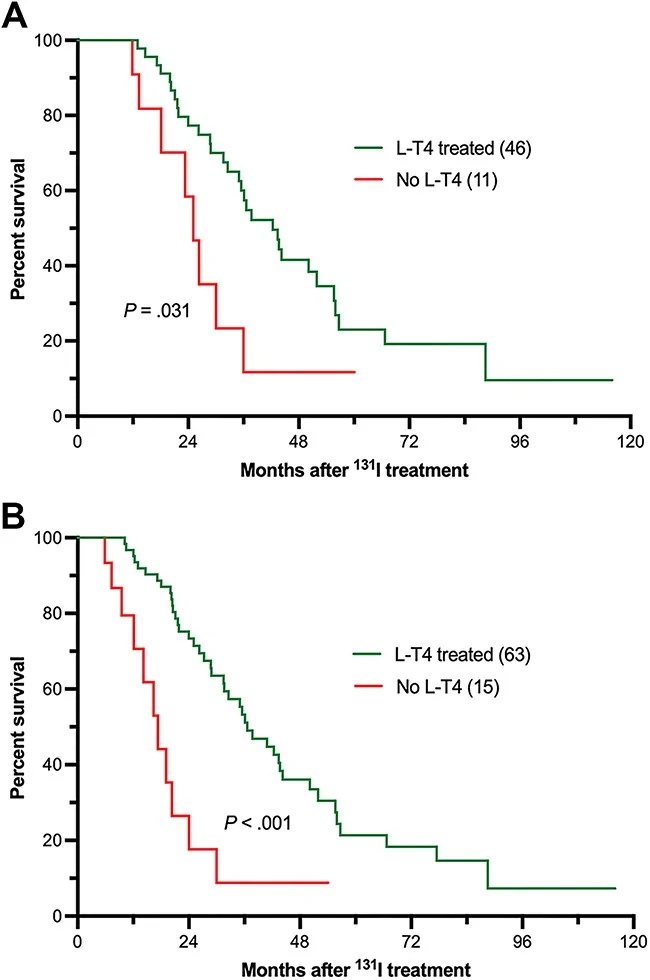
(A) Kaplan–Meier survival curves of 57 cats classified as having overt hypothyroidism at the 6- to 12-month follow-up period after ^131^I treatment, divided into the 46 cats treated with LT4 vs the 11 cats that were not treated. (B) Kaplan–Meier survival curves of 78 cats with overt hypothyroidism, which included all of the cats classified as having overt hypothyroidism at the 6- to 12-month follow-up period, as well as 21 additional cats that developed late-onset overt hypothyroidism. Again, these 78 cats were divided into the 63 cats treated with LT4 vs the 15 cats that were not supplemented with LT4. Abbreviation: LT4 = levothyroxine.

Among the 57 cats classified as overtly hypothyroid at the 6- to 12-month evaluation, 46 received levothyroxine treatment and 11 did not. Kaplan–Meier analysis indicated that levothyroxine-treated cats survived longer than untreated cats (median, 42.3 months; 95% CI, 32.6-51.9 months vs 25.1 months; 95% CI, 13.3-36.0 months; *P* = .031; Figure 3A). Univariable Cox proportional hazards analysis indicated that levothyroxine treatment lowered the hazard of death (Table 3). After adjustment for age, sex, and serum creatinine concentration in the multivariable Cox proportional hazards analysis, however, levothyroxine treatment no longer independently predicted hazard of death (Table 3).

**Table 3:** Cox proportional hazards models for survival in overt hypothyroid cats that were either treated or not treated with levothyroxine.

**Cohort**	**Variable**	**Univariable HR (95% CI)**	***P* value**	**Multivariable HR (95% CI)**	***P* value**
**57-cat cohort**	LT4 treated vs untreated	0.43 (0.19-0.95)	.037	0.46 (0.20-1.03)	.060
	Age, per 1-year increase	1.19 (1.03-1.37)	.020	1.17 (1.00-1.37)	.051
	Male vs female	2.02 (0.97-4.23)	.062	1.77 (0.84-3.74)	.133
	Creatinine, per 1 mg/dL increase	1.77 (0.91-3.44)	.091	1.50 (0.74-3.03)	.261
**78-cat cohort**	LT4 treated vs untreated	0.40 (0.20-0.79)	.009	0.40 (0.20-0.79)	.009
	Age, per 1-year increase	1.20 (1.06-1.36)	.004	1.19 (1.04-1.36)	.010
	Male vs female	1.71 (0.92-3.18)	.088	1.55 (0.83-2.88)	.169
	Creatinine, per 1 mg/dL increase	1.64 (0.94-2.85)	.080	1.50 (0.83-2.70)	.180

HRs < 1.0 indicate lower hazard of death. Reference groups: LT4 treatment = untreated; sex = female.

The 57-cat cohort consisted of the 57 cats classified as having overt hypothyroidism at 6-12 months after ^131^I treatment. The 78-cat cohort also includes these 57 cats, plus 16 cats initially classified as subclinically hypothyroid at 6-12 months that subsequently became overtly hypothyroid, as well as 5 cats classified as euthyroid that subsequently developed late-onset overt hypothyroidism.

Abbreviations: HR = hazard ratio; LT4 = levothyroxine.

A second analysis included an expanded cohort of all 78 cats that developed overt hypothyroidism at any time during follow-up. This group included the 57 cats classified as overtly hypothyroid at 6-12 months, 5 cats initially classified as euthyroid that later developed overt hypothyroidism, and 16 cats initially classified as subclinically hypothyroid that later progressed to overt hypothyroidism. In this expanded overt hypothyroid cohort, 63 cats received levothyroxine treatment and 15 did not. Levothyroxine-treated cats survived longer than untreated cats (median, 36.6 months; 95% CI, 28.9-44.2 months vs 23.3 months; 95% CI, 13.3-30.0 months; *P* < .001; Figure 3B). Both univariable and multivariable Cox proportional hazards analyses indicated that levothyroxine treatment lowered the hazard of death (Table 3). Older age also independently predicted increased hazard of death, whereas sex and serum creatinine concentration did not predict outcome in the multivariable model.

#### Comparison of survival in cats with subclinical hypothyroidism based on levothyroxine treatment status or remission of hypothyroidism

Of the 240 cats with subclinical hypothyroidism, 108 (45%) had remission of hypothyroidism and became euthyroid. Among the remaining 132 cats, 116 (87.9%) remained subclinically hypothyroid and 16 (12.1%) progressed to overt hypothyroidism. Forty-three of these 132 persistently hypothyroid cats (32.6%) were treated with levothyroxine, and 89 were not.

**Figure 4:**
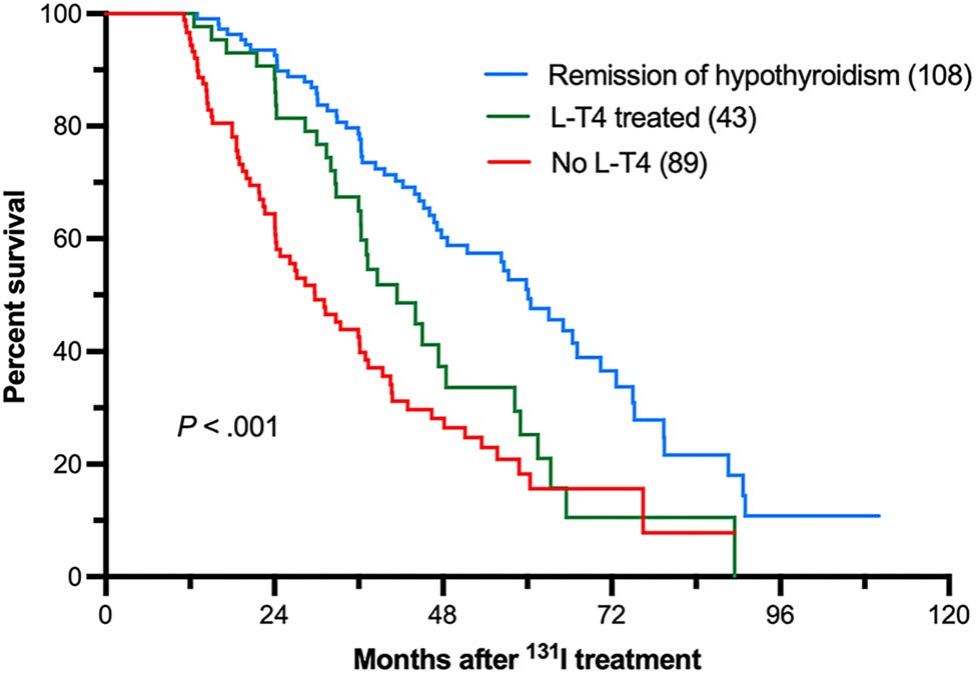
Kaplan–Meier survival curves of 240 cats classified as having subclinical hypothyroidism at the 6- to 12-month follow-up period after ^131^I treatment. Of these, 108 cats underwent remission of hypothyroidism to become either euthyroid (102 cats) or experience relapse of hyperthyroidism (6 cats). Of the remaining 132 cats with persistent hypothyroidism, 43 were treated with levothyroxine and 89 were not treated.

Cats that underwent remission survived longer than both levothyroxine-treated cats and untreated persistently hypothyroid cats (median survival, 60.1 months [95% CI, 47.8-70.4] vs 41.4 months [95% CI, 36.0-48.4] and 29.7 months [95% CI, 24.1-37.0], respectively; Figure 4). Survival did not differ between levothyroxine-treated and untreated persistently hypothyroid cats (*P* = .077).

On multivariable Cox proportional hazards analysis, untreated persistent hypothyroidism independently increased the hazard of death compared with remission of hypothyroidism (hazard ratio [HR], 2.24; 95% CI, 1.52-3.30; *P* < .001; Table 4). In contrast, levothyroxine-treated cats did not have a significant difference in hazard of death compared with cats that underwent remission (HR, 1.52; 95% CI, 0.90-2.56; *P* = .119; Table 4). Older age also independently increased the hazard of death (HR, 1.19 per year; 95% CI, 1.10-1.29; *P* < .001), whereas sex and posttreatment serum creatinine, T4, and TSH concentrations did not independently predict survival.

**Table 4:** Cox proportional hazards models for survival in 240 cats with subclinical hypothyroidism, divided into 108 with remission to become euthyroid, 43 hypothyroid cats treated with levothyroxine, and 89 untreated hypothyroid cats.

**Variable**	**Univariable HR (95% CI)**	***P* value**	**Multivariable HR (95% CI)**	***P* value**
**Thyroid group**				
** Remission of hypothyroidism**	Reference	—	Reference	—
** No LT4 vs remission**	2.75 (1.91-3.95)	<.001	2.24 (1.52-3.30)	<.001
** LT4-treated vs remission**	1.86 (1.20-2.89)	.006	1.52 (0.90-2.56)	.119
**Age (per 1-year increase)**	1.25 (1.16-1.34)	<.001	1.19 (1.10-1.29)	<.001
**Sex (male vs female)**	1.18 (0.84-1.64)	.336	1.29 (0.92-1.81)	.140
**Posttreatment creatinine (per 1-mg/dL increase)**	1.58 (1.22-2.05)	.0006	1.23 (0.91-1.66)	.172
**Posttreatment serum T4 (per 1-μg/dL increase)**	0.74 (0.49-1.13)	.168	1.05 (0.67-1.66)	.826
**Posttreatment serum TSH (per 1-ng/mL increase)**	1.07 (1.01-1.13)	.024	1.01 (0.94-1.08)	.824

HRs > 1.0 indicate increased hazard of death. Remission was the reference group for group comparisons, and female for sex group. The multivariable model included group, age, sex, and posttreatment concentrations of serum T4, TSH, and creatinine. HRs for continuous variables reflect the change in hazard associated with a 1-unit increase in the variable.

Abbreviations: HR = hazard ratio; LT4 = levothyroxine; T4 = thyroxine; TSH = thyroid-stimulating hormone.

#### Comparison of survival in cats based on their final thyroid status at study end-period

Among the 1344 cats, final thyroid classification was euthyroidism in 1037 (77.2%), relapsed hyperthyroidism in 77 (5.7%), untreated subclinical hypothyroidism in 117 (8.7%), levothyroxine-treated subclinical hypothyroidism in 37 (2.8%), untreated overt hypothyroidism in 14 (1.0%), and levothyroxine-treated overt hypothyroidism in 62 (4.6%).

**Figure 5:**
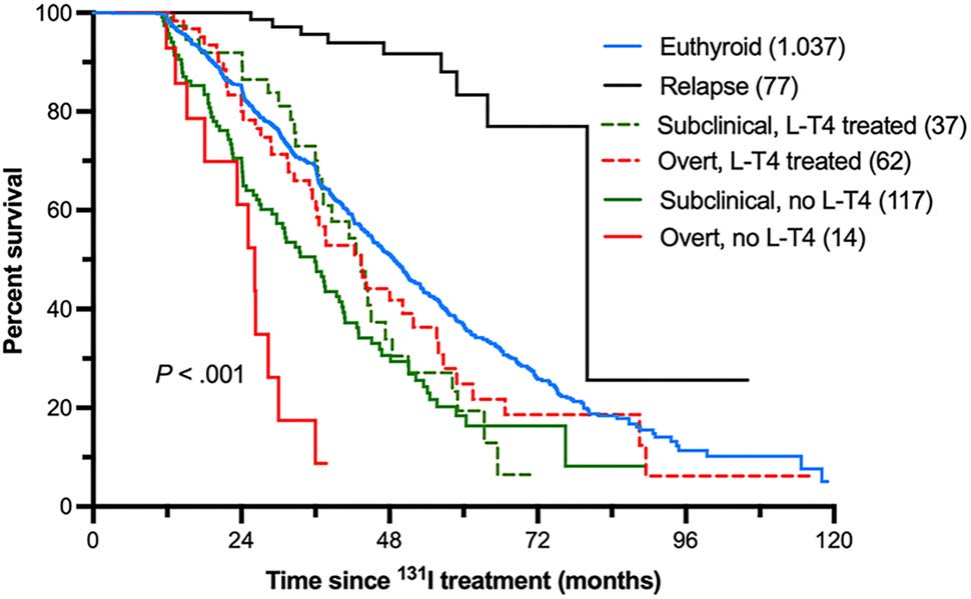
Kaplan–Meier survival curves of 1344 cats grouped according to thyroid status at their final follow-up evaluation. The 6 thyroid outcomes included euthyroidism (1037 cats); relapse of hyperthyroidism (77 cats); subclinical hypothyroidism, either treated with LT4 (37 cats) or not treated with levothyroxine (117 cats); and overt hypothyroidism, either treated with LT4 (62 cats) or not treated (14 cats). Abbreviation: LT4 = levothyroxine.

Survival differed among the 6 final thyroid outcome groups (*P* < .0001; Figure 5). Euthyroid cats lived longer (median, 48.7 months; 95% CI, 45.6-51.0 months) than untreated subclinically hypothyroid cats (median, 35.9 months; 95% CI, 29.0-40.3 months; *P* < .0001) and untreated overtly hypothyroid cats (median, 26.2 months; 95% CI, 15.2-30.0 months; *P* < .0001; Figure 5). In contrast, euthyroid cats did not differ in survival from levothyroxine-treated subclinically hypothyroid cats (median, 43.4 months; 95% CI, 36.3-48.4 months; *P* = .082) or levothyroxine-treated overtly hypothyroid cats (median, 43.4 months; 95% CI, 35.5-51.9 months; *P* = .219; Figure 5). Among cats with overt hypothyroidism, those treated with levothyroxine lived longer than untreated cats (median, 43.4 months; 95% CI, 35.5-51.9 months vs 26.2 months; 95% CI, 15.2-30.0 months; *P* < .0001). Cats that developed recurrent hyperthyroidism had the longest survival (median, 84.2 months), exceeding that of euthyroid cats and all levothyroxine-treated and untreated hypothyroid groups (*P* < .0001; Figure 5).

In univariable Cox proportional hazards analysis, compared with euthyroid cats, the hazard of death was increased in cats with either overt or subclinical hypothyroidism not treated with levothyroxine (Table 5). Overt hypothyroid cats not treated with levothyroxine had the greatest increase in hazard of death. Cats that relapsed had a lower hazard of death than euthyroid cats, likely reflecting the much longer survival time required to develop recurrent hyperthyroidism.

**Table 5:** Cox proportional hazards models for survival in 1344 cats divided into 6 final thyroid groups (euthyroid, relapse, and overt and subclinical hypothyroid, either treated with levothyroxine or not).

**Variable**	**Univariable HR (95% CI)**	***P* value**	**Multivariable HR (95% CI)**	***P* value**
**Group**				
	Reference	—	Reference	—
** Subclinical, untreated vs euthyroid**	1.78 (1.42-2.24)	<.0001	1.47 (1.17-1.86)	.001
** Subclinical, LT4-treated vs euthyroid**	1.39 (0.95-2.04)	.088	1.08 (0.72-1.60)	.722
** Overt, untreated vs euthyroid**	4.50 (2.47-8.22)	<.0001	4.06 (2.22-7.44)	<.0001
** Overt, LT4-treated vs euthyroid**	1.22 (0.89-1.66)	.218	1.11 (0.81-1.54)	.511
** Relapse vs euthyroid**	0.18 (0.10-0.33)	<.0001	0.21 (0.11-0.40)	<.0001
**Age (per 1-year increase)**	1.24 (1.20-1.28)	<.0001	1.20 (1.16-1.24)	<.0001
**Sex (male vs female)**	1.30 (1.13-1.50)	.0002	1.37 (1.19-1.58)	<.0001
**Posttreatment creatinine (per 1-mg/dL increase)**	1.61 (1.41-1.84)	<.0001	1.25 (1.08-1.45)	.003
**Posttreatment serum T4 (per 1-μg/dL decrease)**	0.80 (0.71-0.90)	.0002	0.91 (0.80-1.04)	.156
**Posttreatment serum TSH (per 1-ng/mL increase)**	1.04 (1.00-1.07)	.027	0.99 (0.94-1.03)	.566

HRs > 1.0 indicate increased hazard of death; HRs < 1.0 indicate lower hazard of death.

Euthyroid cats were the reference group for group comparisons. The multivariable model included group, age, sex, and posttreatment serum creatinine, T4, and TSH concentrations at 6-12 months.

HRs for continuous variables reflect the change in hazard associated with a 1-unit increase or decrease in the variable.

Abbreviations: HR = hazard ratio; LT4 = levothyroxine; T4 = thyroxine; TSH = thyroid-stimulating hormone.

In the multivariable Cox proportional hazards model adjusted for age, sex, and posttreatment serum concentrations of creatinine, T4, and TSH, both untreated subclinical and overt hypothyroid groups remained independently associated with an increased hazard of death, compared with euthyroid cats (Table 5). In contrast, neither levothyroxine-treated subclinical nor overt hypothyroidism was associated with a different hazard of death compared with euthyroidism. Increasing age, male sex, and higher posttreatment serum creatinine concentration were also independently associated with an increased hazard of death.

## Discussion

This large study evaluates long-term thyroid outcomes and survival in hyperthyroid cats successfully treated with ^131^I. Previous studies of ^131^I treatment have generally focused on short-term cure rates, thyroid status during the first several months after treatment, or survival in smaller cohorts.^3–19^ In contrast, the present study followed cats for up to 12 years after treatment and evaluated not only survival but also subsequent changes in thyroid status after the initial 6- to 12-month posttreatment classification. Several clinically important findings emerged. First, most cats classified as euthyroid 6-12 months after ^131^I treatment remained euthyroid long term; however, a minority subsequently developed either late-onset subclinical or overt hypothyroidism, or recurrent hyperthyroidism. Second, subclinical hypothyroidism was often transient, with nearly half of affected cats becoming euthyroid during follow-up. Finally, persistent, untreated overt or subclinical hypothyroidism was associated with shorter survival, especially when cats were classified according to final thyroid outcome. In contrast, survival of levothyroxine-treated cats with either overt or subclinical hypothyroidism did not differ significantly from that of euthyroid cats in these models. These findings support continued monitoring of thyroid status after ^131^I treatment and levothyroxine supplementation in cats that develop overt hypothyroidism or persistent, progressively worsening subclinical hypothyroidism.

The finding that almost 90% of cats classified as euthyroid 6-12 months after ^131^I treatment remained euthyroid throughout follow-up supports the long-term effectiveness of ^131^I treatment. However, euthyroidism at the 6- to 12-month evaluation did not guarantee lifelong normality of thyroid function. A small proportion of initially euthyroid cats developed late-onset hypothyroidism (usually subclinical, but sometimes overt), whereas a slightly larger proportion developed recurrent hyperthyroidism. These findings indicate that clinicians should continue to reevaluate thyroid function at 6- to 12-month intervals, even after apparent biochemical cure. This is particularly important if clinical signs recur, body weight changes, indicators of renal function worsen, or serum T4 or TSH concentrations drift toward the limits of their reference intervals.

Late-onset hypothyroidism developed in 43 cats initially classified as euthyroid, most often within the first 1-2 years after treatment, although hypothyroidism developed as late as 48 months after ^131^I treatment. Older age, lower serum T4 concentration, and higher serum TSH concentration at the 6- to 12-month evaluation were associated with an increased risk for subsequent hypothyroidism. Cats with low-normal T4 concentrations and high-normal or increased TSH concentrations after treatment may have limited thyroid reserve, even before they meet criteria for overt or subclinical hypothyroidism.^21^^,^^22^^,^^28^ In such cats, progressive loss of functional thyroid tissue associated with delayed radiation effects may contribute to later hypothyroidism.^29–31^

Recurrent hyperthyroidism was uncommon but not rare, occurring in 70 (6.7%) of 1047 initially euthyroid cats. Median time to relapse was almost 4 years after treatment, and relapse was associated with younger age, higher posttreatment serum T4 concentration, and longer time since ^131^I treatment. These findings suggest that relapse is usually a late event and most likely reflects the progressive nature of feline nodular thyroid disease. Early relapse, particularly within 2 years after treatment, may reflect continued growth of residual autonomous thyroid tissue that was not completely ablated by ^131^I. In contrast, later relapse, especially beyond 3 years after treatment, may be more consistent with development of new hyperfunctioning adenomatous nodules in previously nonautonomous thyroid tissue.^32–34^ The occurrence of late relapse also parallels observations in human patients with autonomous thyroid nodules or toxic nodular goiter, in whom recurrent hyperthyroidism can occur after apparently successful radioiodine treatment.^35–37^

Cats classified as subclinically hypothyroid at 6-12 months commonly underwent remission of hypothyroidism to become euthyroid. Of 240 cats, 102 became euthyroid, with a median time to remission of 20.3 months. This finding supports previous observations that some hyperthyroid cats develop transient rather than permanent hypothyroidism after successful radioiodine treatment.^38^ The mechanism for this delayed recovery is uncertain, but remission may reflect gradual recovery or compensatory hypertrophy of residual normal thyroid tissue that was not destroyed by ^131^I, delayed recovery from radiation-induced thyroid injury, or sufficient functional reserve in partially damaged thyroid tissue to restore euthyroidism over time. With subclinical hypothyroidism, the normal serum T4 concentration indicates that some thyroid hormone production persists, but the increased serum TSH concentration suggests that this production is maintained only under increased pituitary stimulation. Our findings suggest that cats with lower TSH concentrations (<1.5 ng/mL) at the 6- to 12-month evaluation may have greater residual thyroid reserve and a greater likelihood of subsequent remission. In cats with TSH concentrations < 1.5 ng/mL and stable renal function, we recommend initial monitoring without levothyroxine supplementation. In contrast, levothyroxine supplementation should be strongly considered in cats with higher or progressively increasing TSH concentrations, declining T4 concentrations, new or worsening azotemia, or clinical signs compatible with hypothyroidism.

Conversely, cats classified as overtly hypothyroid at 6-12 months rarely underwent remission of hypothyroidism. Overt hypothyroidism after ^131^I treatment likely reflects more extensive destruction of both adenomatous and normal thyroid tissue, resulting in markedly insufficient thyroid hormone production despite markedly increased pituitary TSH stimulation.^39^^,^^40^ Because untreated overt hypothyroidism can contribute to shorter survival, worsening azotemia, and clinical signs such as lethargy, weight gain, and dermatologic abnormalities, levothyroxine supplementation should be strongly considered in all of these cats. Continued monitoring remains important after supplementation is started, however, as thyroid hormone requirements may change over time.^41^ Rarely, cats may recover sufficient thyroid function to allow dose reduction or discontinuation of levothyroxine.

When survival was compared according to thyroid status at 6-12 months after ^131^I treatment, overtly hypothyroid cats survived for a shorter time than euthyroid cats, whereas survival in cats with subclinical hypothyroidism fell between these groups and was not different from either. However, after adjustment for relevant demographic and clinicopathologic variables, thyroid status was no longer independently associated with survival. Instead, older age, male sex, and higher serum creatinine concentration were independently associated with increased hazard of death. These findings suggest that the apparent survival disadvantage in overtly or subclinically hypothyroid cats was largely attributable to other adverse prognostic factors rather than to thyroid status alone. Older age and male sex have also been associated with shorter survival in other studies of ^131^I-treated hyperthyroid cats.^8^^,^^11^^,^^14^^,^^17^^,^^42^

Models based on final thyroid status at study end provided additional clinical insight. When cats were classified by final thyroid status and levothyroxine treatment status, untreated overt and subclinical hypothyroidism remained independently associated with increased hazard of death compared with euthyroidism. In contrast, survival of levothyroxine-treated cats with either overt or subclinical hypothyroidism did not differ from euthyroid cats (Figure 5). These findings suggest that persistent untreated overt hypothyroidism, as well as persistent or progressively worsening subclinical hypothyroidism, may adversely affect long-term outcome, and that levothyroxine supplementation might mitigate this effect. This interpretation is consistent with previous evidence that iatrogenic hypothyroidism, particularly when accompanied by azotemia, can reduce survival after treatment of hyperthyroidism.^21^^,^^43^^,^^44^

These 2 approaches to survival analysis address different clinical questions. The 6- to 12-month thyroid classification reflects the first standardized posttreatment outcome and is therefore useful for prognosis and client counseling at the time clinicians usually assess treatment response. In contrast, final thyroid status at study end reflects what thyroid function ultimately became during long-term follow-up and better captures the clinical consequences of persistent overt or subclinical hypothyroidism, recurrent hyperthyroidism, remission of subclinical hypothyroidism, and levothyroxine treatment. Because thyroid status after ^131^I treatment is dynamic, both analyses provide complementary information.

Renal function remains central to clinical management after ^131^I treatment and should be monitored together with serum T4 and TSH concentrations.^21^^,^^22^ Hyperthyroidism increases renal blood flow and glomerular filtration rate (GFR), potentially masking CKD, whereas successful treatment decreases GFR and can unmask renal dysfunction.^12^^,^^45–47^ Iatrogenic hypothyroidism may further reduce GFR, increase serum creatinine concentration, and contribute to azotemia.^21^^,^^43^^,^^45^^,^^48^ In the present study, higher posttreatment serum creatinine concentration was consistently associated with shorter survival, reinforcing the need to interpret renal values in the context of thyroid status.^49^ In hypothyroid cats (either subclinical or overt) that become azotemic after treatment, levothyroxine supplementation may improve renal perfusion and lessen or resolve azotemia in some cases, although persistent CKD remains in others.^21^^,^^48^

Renal function might also influence the likelihood of developing ^131^I-induced hypothyroidism. In a previous study, hyperthyroid cats with concurrent or masked CKD appeared at greater risk for iatrogenic hypothyroidism than cats with normal renal function.^48^ Because stable iodine and ^131^I are eliminated primarily by the kidneys, impaired renal clearance may prolong radioiodine retention,^50–53^ potentially increasing thyroid radiation exposure and the risk of hypothyroidism. Although the present study was not designed to evaluate renal function as a determinant of ^131^I kinetics, the associations among serum creatinine concentration, hypothyroidism, and survival support the broader concept that renal status affects both treatment outcome and long-term prognosis.

The survival findings in overtly hypothyroid cats further support a potential benefit of levothyroxine supplementation, although interpretation requires caution. Among the 57 cats classified as overtly hypothyroid at 6-12 months, levothyroxine-treated cats had longer median survival than untreated cats, but treatment did not independently predict survival after adjustment for other variables (Figure 3A). In the expanded cohort of 78 cats, which included euthyroid cats with late-onset overt hypothyroidism and cats that progressed from subclinical to overt hypothyroidism, levothyroxine-treated cats survived longer than untreated cats, even after adjustment for other variables (Figure 3B; Table 3). The stronger association in this expanded cohort likely reflects both the larger number of overtly hypothyroid cats and the fact that this analysis evaluated the clinical association of overt hypothyroidism whenever it developed during follow-up, rather than only among cats overtly hypothyroid at the standardized 6- to 12-month evaluation. Because survival in this expanded cohort was calculated from the time overt hypothyroidism was diagnosed, this analysis also avoided assigning survival time before overt hypothyroidism had actually developed. However, differences in the timing or severity of hypothyroidism, as well as confounding by clinical decision-making, also could have contributed to the observed survival difference. Nevertheless, the consistent finding of longer survival in treated cats, especially in the final-outcome analysis, strongly supports levothyroxine supplementation for cats with overt hypothyroidism.

The findings in cats with subclinical hypothyroidism are more nuanced. Cats whose subclinical hypothyroidism resolved had the longest survival, whereas cats with persistent untreated subclinical hypothyroidism had the shortest survival (Figure 4). Survival in levothyroxine-treated cats fell between these 2 groups and, in multivariable analysis, did not differ from that of cats whose subclinical hypothyroidism resolved. This pattern suggests that spontaneous remission identifies a subgroup with greater thyroid reserve and better overall prognosis. Conversely, persistent untreated subclinical hypothyroidism might not be benign, particularly if associated with new or worsening azotemia, progressive serum TSH elevation, declining T4 concentration, or clinical signs compatible with hypothyroidism. Our results do not indicate that every cat with subclinical hypothyroidism requires immediate levothyroxine treatment but rather support the use of serial monitoring and individualized treatment. However, given the favorable safety profile of levothyroxine^54^ and the fact that remission of subclinical hypothyroidism may take many months, a therapeutic trial of levothyroxine is reasonable in selected cats. In such cases, levothyroxine can be discontinued after several months, with reevaluation of serum T4 and TSH concentrations to determine whether continued supplementation is needed or remission has occurred.

The present findings are particularly relevant when compared with the recent study by Cox et al.^44^ In that study of 117 ^131^I-treated cats, thyroid status was assessed by TSH stimulation testing rather than serum TSH measurements, and cats were classified as euthyroid, equivocal, or hypothyroid. Euthyroid azotemic cats and untreated hypothyroid cats had shorter survival than euthyroid nonazotemic cats. In addition, levothyroxine-supplemented nonazotemic hypothyroid cats had longer survival than untreated nonazotemic hypothyroid cats, whereas supplementation was not associated with longer survival in azotemic hypothyroid cats.^44^ These findings are broadly compatible with the present study: persistent untreated hypothyroidism was associated with poorer survival, whereas levothyroxine-treated hypothyroid cats generally had survival more similar to euthyroid cats. However, the present study included a much larger cohort, used longitudinal serum T4 and TSH concentrations to reclassify thyroid status over time, and distinguished overt hypothyroidism, subclinical hypothyroidism, remission of subclinical hypothyroidism, late-onset hypothyroidism, and relapse. In contrast, Cox et al. used one-time TSH stimulation testing, included fewer hypothyroid cats, and had limited power after stratification by azotemic status and levothyroxine treatment. Taken together, both studies support the clinical relevance of persistent untreated hypothyroidism but also indicate why survival results may differ among studies depending on renal status, when thyroid function is assessed, and how hypothyroidism is defined.

This study has several limitations. Although the cohort was large and follow-up was prolonged, levothyroxine treatment was not randomized. Treatment decisions were made by owners and referring veterinarians, creating potential selection bias and confounding by indication. Treated and untreated cats may have differed in disease severity, follow-up frequency, comorbidities, owner commitment, or perceived prognosis. Causes of death or euthanasia were not uniformly available, so survival was evaluated as all-cause mortality. In addition, most cats were followed by referring veterinarians, which may have introduced variability in the timing and completeness of follow-up. Analyses involving remission of subclinical hypothyroidism also may be affected by survivor bias, because remission could only be documented in cats that survived long enough to undergo repeated thyroid testing. Although multivariable models adjusted for major covariates, residual confounding by unmeasured factors such as blood pressure, proteinuria, body or muscle condition, CKD severity, concurrent disease, and owner treatment preferences cannot be excluded.

Despite these limitations, our study has important strengths. The cohort size is substantially larger than those in previous survival studies of ^131^I-treated cats. Thyroid status was classified using both serum T4 and TSH concentrations, allowing distinction between overt and subclinical hypothyroidism. Prolonged follow-up allowed identification of late-onset hypothyroidism (both overt and subclinical), remission of subclinical hypothyroidism, and relapse of hyperthyroidism. Finally, survival analyses assessed whether thyroid status remained associated with outcome after adjustment for clinically relevant covariates.^23^^,^^25^^,^^26^

In conclusion, thyroid status after radioiodine treatment of hyperthyroid cats is dynamic and requires periodic, long-term monitoring. Most cats that are euthyroid at 6-12 months remain euthyroid, but some later develop subclinical or overt hypothyroidism, or recurrent hyperthyroidism. Subclinical hypothyroidism frequently resolves, whereas overt hypothyroidism is usually persistent. Initial posttreatment hypothyroidism is associated with shorter crude survival, but this association appears largely explained by age, sex, and renal function. In contrast, persistent untreated overt or subclinical hypothyroidism is associated with an increased hazard of death, whereas levothyroxine-treated hypothyroid cats have survival more similar to euthyroid cats. These findings support continued long-term monitoring of serum T4, TSH, and renal function after ^131^I treatment and individualized use of levothyroxine supplementation in cats with persistent hypothyroidism, especially when accompanied by worsening azotemia, increasing TSH concentration, declining T4 concentration, or clinical signs compatible with hypothyroidism.

## Off-label antimicrobial declaration

The authors declare no off-label use of antimicrobials.

## Institutional animal care and use committee or other approval declaration

The authors declare no institutional animal care and use committee or other approval was needed.

## Appendix

### Materials and methods: statistics

All data were assessed for normality by the D’Agostino–Pearson test and by visual inspection of graphical plots.^55^ Data were not normally distributed; therefore, all analyses were performed using nonparametric tests.

Comparisons between 2 continuous variables between groups were analyzed with the Mann–Whitney *U* test. Comparisons among 3 continuous variables were analyzed with the Kruskal–Wallis test, followed by Dunn’s multiple comparisons test.^56^ Categorical variables between groups were compared by the χ^2^ test or Fisher’s exact test, followed by the Holm–Bonferroni correction procedure for within group comparison, as appropriate.^57^^,^^58^ Multivariable logistic regression was used to identify factors associated with remission of hypothyroidism to become euthyroid and with relapse of hyperthyroidism.^59^

Kaplan–Meier analysis was used to estimate the cumulative incidence of recurrent hyperthyroidism and late-onset hypothyroidism after ^131^I treatment.^25^^,^^60^ For recurrent hyperthyroidism, the event was defined as development of recurrent hyperthyroidism after initial successful treatment. Cats that did not develop recurrent hyperthyroidism were censored at the date of last follow-up, death, or last known euthyroid status. For late-onset hypothyroidism, the event was defined as development of hypothyroidism after initial euthyroid classification. Cats that did not develop late-onset hypothyroidism were censored at the date of last follow-up, death, or last known euthyroid status. Time-to-event was calculated in months from the date of ^131^I treatment to the first documented occurrence of recurrent hyperthyroidism or late-onset hypothyroidism. Kaplan–Meier curves were plotted as 1 minus the Kaplan–Meier survival estimate to display the cumulative proportion of cats developing each outcome over time. Censoring was indicated on the curves. Median time to event and 95% CIs were reported when estimable.

Survival time was defined as the interval from treatment with radioactive iodine (^131^I) to death or euthanasia. Cats that were alive at the end of follow-up or lost to follow-up were right-censored at the date of last contact. Survival analyses were performed to compare outcomes among cats grouped according to thyroid status at the 6- to 12-month follow-up evaluation and, in additional analyses, according to subsequent thyroid outcome subgroups, including levothyroxine treatment status where applicable. Kaplan–Meier curves were constructed for each comparison, and survival distributions were compared by log-rank testing.

Univariable Cox proportional hazards regression was first used to estimate the association of each predictor with survival. Multivariable Cox proportional hazards regression then was used to assess whether thyroid group was independently associated with survival after adjustment for selected covariates.^26^ Models were constructed using forced entry of clinically relevant variables, with thyroid outcome group retained as the primary exposure variable. Depending on the specific analysis, candidate covariates included age, sex, breed, body weight, serum creatinine concentration, pretreatment serum T4 concentration, and posttreatment serum T4 and TSH concentrations; final multivariable models retained the covariates specified for each comparison. Categorical variables were entered with prespecified reference groups, and continuous variables were modeled as continuous predictors. Hazard ratios and 95% CIs were calculated. The proportional-hazards assumption was evaluated for each Cox model using Schoenfeld residuals in relation to ranked event time, together with variable-specific testing for nonproportionality.^27^ A 2-sided *P* value ≤ .05 was considered significant.

## Abbreviations

CKD: chronic kidney disease
HR: hazard ratio
OR: odds ratio
